# Supplementary material for: Pathobiont-induced suppressive immune imprints thwart T cell vaccine responses
Source: Nat Commun. 2024 Dec 16;15:10335. doi: 10.1038/s41467-024-54644-w (PMC11649901; doi:10.1038/s41467-024-54644-w)
Supplement: Supplementary file 1 — Supplementary Information [file 41467_2024_54644_MOESM1_ESM.pdf]

Pathobiont-induced suppressive immune imprints thwart T cell vaccine responses

Irshad Ahmed Hajam[1], Chih-Ming Tsai[1], Cesia Gonzalez[1], Juan Raphael Caldera[1,2], María Lázaro Díez[1,3], Xin Du[1], April Aralar[1], Brian Lin[1], William Duong[1], George Y. Liu[1,4]

[1]Department of Pediatrics, University of California San Diego, San Diego, CA 92093, USA

[2]Quest Diagnostics, 33608 Ortega Hwy., San Juan Capistrano, CA 92675

[3]AIDS Research Institute (IrsiCaixa). VIRus Immune Escape and VACcine Design (VIRIEVAC) Universitary Hospital German Trias i Pujol Crta Canyet s/n 08916, Badalona, Barcelona, Spain.

[4]Division of Infectious Diseases, Rady Children's Hospital, San Diego, CA 92123, USA

Corresponding author: George Y. Liu (gyliu@health.ucsd.edu)

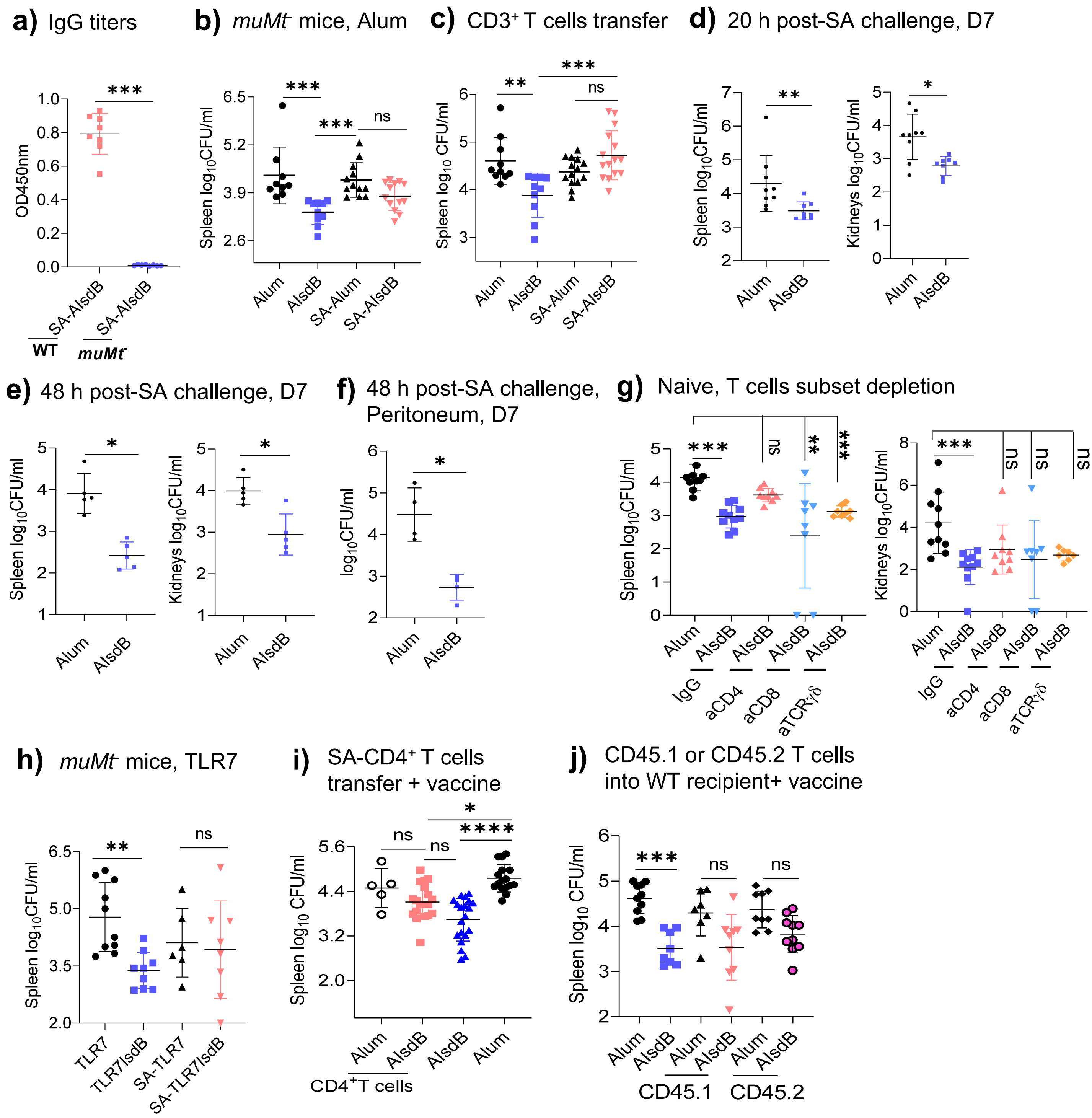
a) IgG titers
OD450nm
SA-AlsdB
SA-AlsdB
WT
muMt-
b) muMt- mice, Alum
Spleen log10CFU/ml
Alum
AlsdB
SA-Alum
SA-AlsdB
c) CD3+ T cells transfer
Spleen log10 CFU/ml
d) 20 h post-SA challenge, D7
Kidneys log10CFU/ml
e) 48 h post-SA challenge, D7
f) 48 h post-SA challenge, Peritoneum, D7
log10CFU/ml
g) Naive, T cells subset depletion
IgG
aCD4
aCD8
aTCRγδ
h) muMt- mice, TLR7
TLR7
TLR7IsdB
SA-TLR7
SA-TLR7IsdB
i) SA-CD4+ T cells transfer + vaccine
CD4+T cells
j) CD45.1 or CD45.2 T cells into WT recipient+ vaccine
CD45.1
CD45.2
ns

Supplementary Fig. 1 Prior SA exposure abrogates vaccine-induced protective T cell responses.

a) Serum from SA-exposed WT or *muMt*$^{-}$ AIsdB vaccinated mice was isolated on 7 dpv and analyzed for IsdB titers by an indirect ELISA (n=8 per group).

b) Naïve or SA-exposed *muMt*$^{-}$ mice were vaccinated with either Alum (n=9 for alum and n=12 for SA-Alum) or AIsdB (n=10 for AIsdB and n=13 for SA-AIsdB), then SA challenged as in **Figure 1a**.

c) Naïve or SA-exposed WT mice were vaccinated with either Alum (n=10 for alum and n=14 for SA-Alum) or AIsdB (n=10 for AIsdB and n=15 for SA-AIsdB) as in **Figure 1a**. Splenic $CD3^+$ T cells isolated 14 dpv were adoptively transferred into naïve recipient mice, followed by SA challenge**.**

d) Naïve WT mice were vaccinated with either Alum (n=9) or AIsdB (n=8), then SA challenged as in Figure 1**a** (harvest of bacteria 20 h later).

e-f) Naïve mice were vaccinated with either Alum (n=5 in **e** and 4 in **f**) or AIsdB (n=5 **in e** and 4 in **f**) as in main **Figure 1a**, then SA challenged. Bacterial burden in spleen, kidneys, and peritoneum at 48 h post SA challenge.

g) Naive mice were vaccinated with either Alum (n=10) or AIsdB as in **Figure1 a**, then one day before and on the day of SA challenge, were treated with isotype IgG (n=10), anti-CD4 (n=9), anti-CD8 (n=8) or anti-TCR γδ (n=8) antibodies, then challenged with SA.

h) Naïve or SA-exposed *muMt*$^{-}$ mice were vaccinated with either TLR7 alone (n=10 for TLR7 and n=6 for SA-TLR7) or TLR7IsdB (n=9 for TLR7IsdB and n=8 for SA-TLR7), then SA challenged as in **Figure 1a**.

i) SA-exposed splenic $CD4^+$ T cells ($1x10^7$) were adoptively transferred into naïve recipient WT mice. One day later, the recipient mice were vaccinated with either Alum (Alum-CD4, n=5; Alum, n=16) or AIsdB (AIsdb-CD4, n=19 and AIsdB, n=20), and challenged with SA as in **Figure 1e**.

j) SA-exposed splenic CD45.1 $CD4^+$ T cells ($1x10^7$) were transferred into naïve CD45.2 mice followed by AIsdB vaccination. Then, splenic CD45.1 or CD45.2 $CD4^+$ T cells were isolated 7 dpv and transferred into naive recipients, followed by vaccination and SA challenge of the recipients as in main **Figure 1g**. Alum (n=10,7 and 9 respectively). AIsdB (8, 9, and 10 respectively).

Data were from one to four independent experiments with each data point representing one mouse. The data is presented as mean ± SD of biological replicates. The data in **a, e-f** were analyzed by two-tailed non-parametric Mann-Whitney T test, while the data in **b-d, g-j** were analyzed by Kruskal-Wallis non-parametric one-way ANOVA test. *p<0.05, **p<0.01, ***p<0.001, ****p<0.0001. ns-non-significant. D, day. dpv*,* days post-last vaccination. SA, *Staphylococcus aureus.* WT, wild-type. Source data are provided as Source Data File.

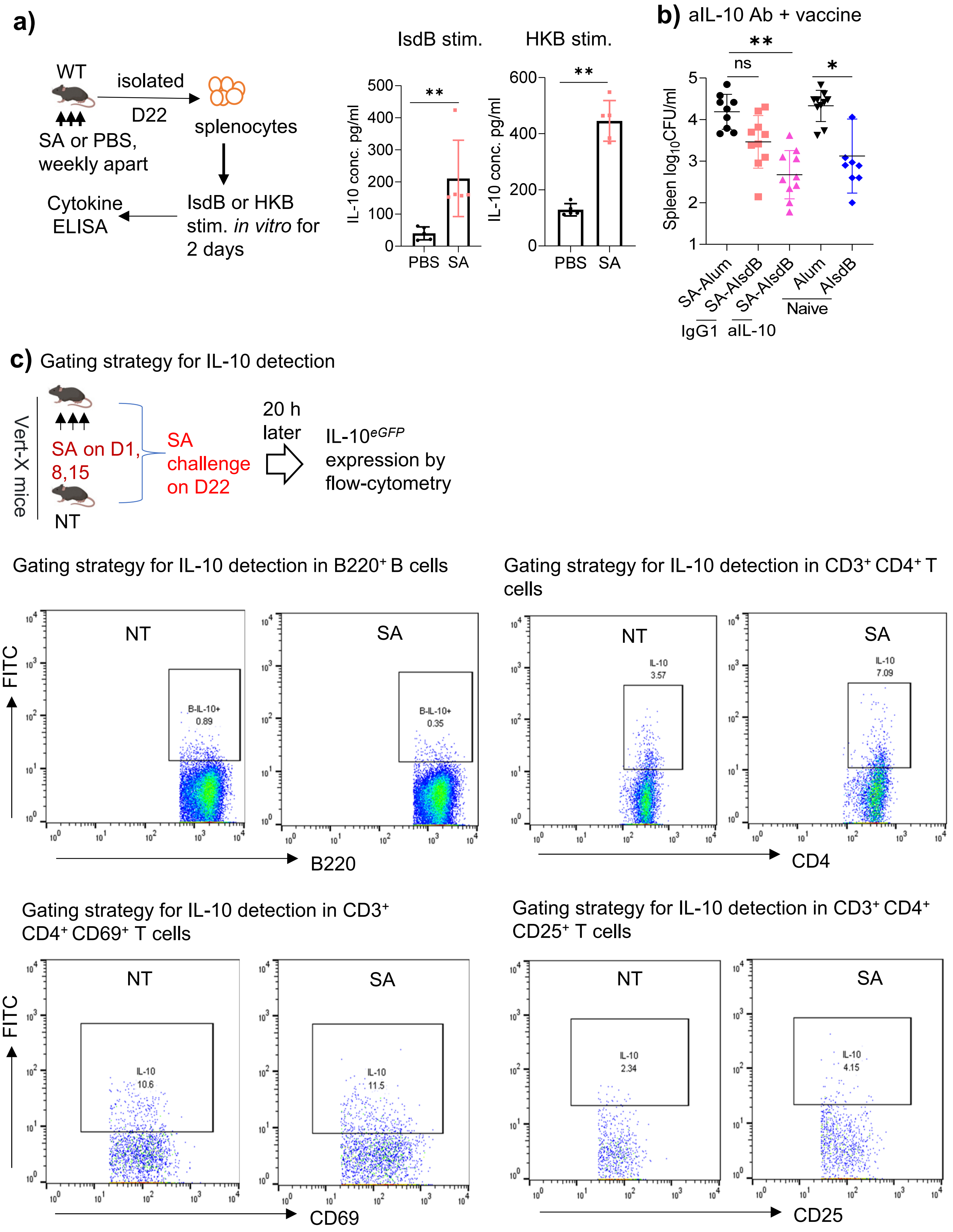
a)
WT
isolated
D22
splenocytes
SA or PBS, weekly apart
IsdB or HKB stim. in vitro for 2 days
Cytokine ELISA
IsdB stim.
HKB stim.
IL-10 conc. pg/ml
PBS
SA
**
b) aIL-10 Ab + vaccine
Spleen log10CFU/ml
ns
**
*
SA-Alum
SA-AIsdB
SA-AIsdB
Alum
AIsdB
Naive
IgG1
aIL-10
c) Gating strategy for IL-10 detection
Vert-X mice
SA on D1, 8,15
NT
SA challenge on D22
20 h later
IL-10eGFP expression by flow-cytometry
Gating strategy for IL-10 detection in B220+ B cells
NT
SA
B-IL-10+
0.89
0.35
FITC
B220
Gating strategy for IL-10 detection in CD3+ CD4+ T cells
IL-10
3.57
7.09
CD4
Gating strategy for IL-10 detection in CD3+ CD4+ CD69+ T cells
10.6
11.5
CD69
Gating strategy for IL-10 detection in CD3+ CD4+ CD25+ T cells
2.34
4.15
CD25

Supplementary Fig. 2 IL-10 plays a critical role in vaccine suppression

a) WT mice were either treated with PBS (n=5) or SA (n=5, 3x, 3x10^7CFU) and splenocytes (pooled, n=5, 1x10^6) were isolated on D7 post-SA exposure and were stimulated with either IsdB (10 µg/ml) or HKB (1:10). After 60 h of stimulation, culture supernatants were analyzed for IL-10.

b) Naïve (n=10 for Alum and n=9 for AIsdB) or SA-exposed mice were vaccinated with AIsdB with (n=10) or without anti-IL-10 MAb (n=10) before and after vaccination, then challenged with SA (n=9-10 per group). SA-Alum, n=9.

c) Experimental setting, and gating strategy for the measurement of IL-10 expression by adaptive immune cells. Vert-X mice were left untreated (NT) or exposed to SA (3x10^7CFU), then challenged with SA. *eGFP* (IL-10) expression in splenocytes was analyzed by flow cytometry after 20 h.

Data were from one to two independent experiments with each data point representing one mouse in **b.** The data is presented as mean ± SD of biological replicates., except in **a** (data is presented as mean ± SD of five technical replicates). The data in **a** was analyzed by two-tailed non-parametric Mann-Whitney T test, and the data in **b** by Kruskal-Wallis non-parametric one-way ANOVA test. *p<0.05, **p<0.001. D, day. HKB, heat-killed bacteria. SA, *Staphylococcus aureus*. WT-wild-type. Source data are provided as Source Data File. . Mouse image was created by BioRender (Created in BioRender. Hajam, I. (2024) https://BioRender.com/a18v205).

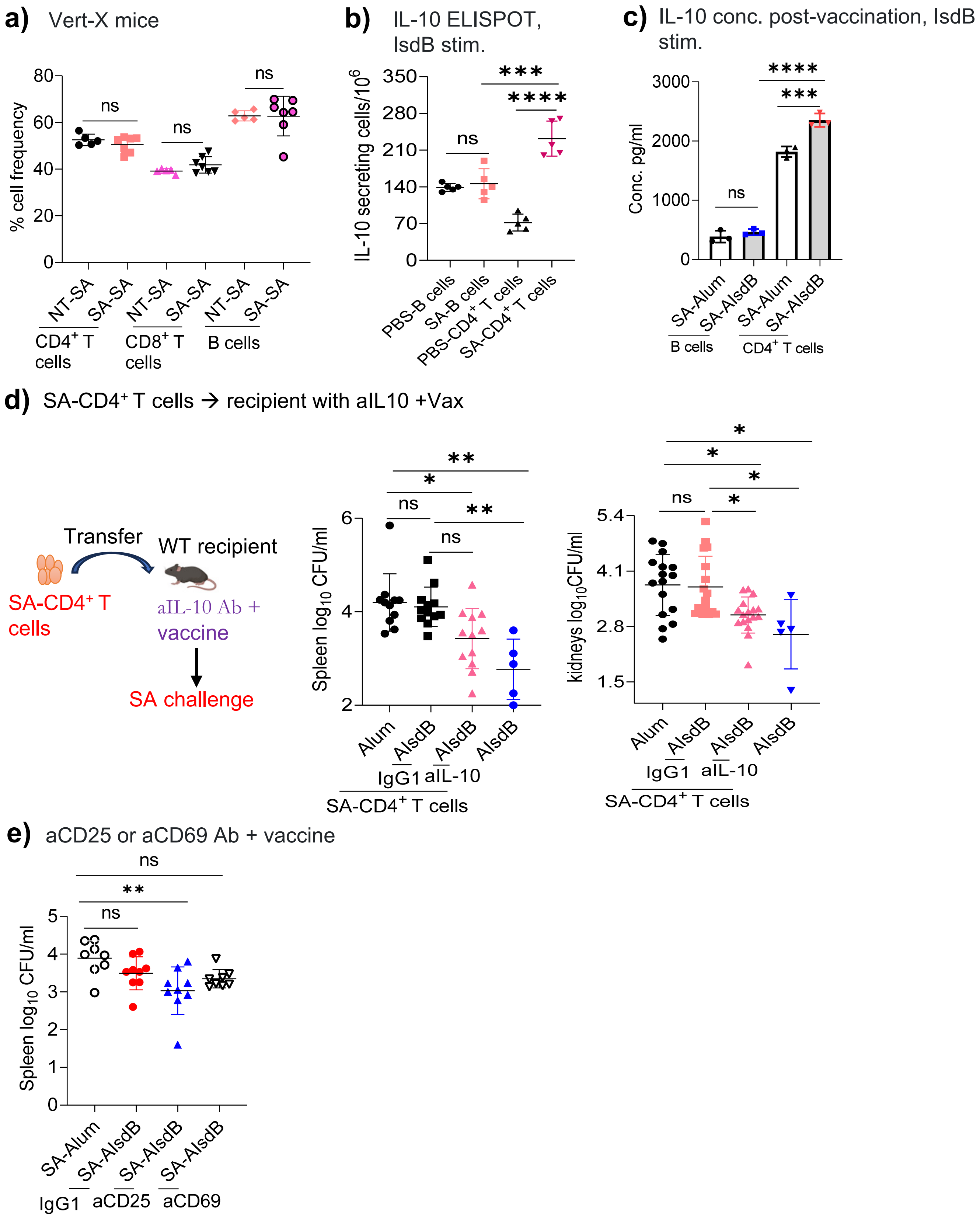

a) Vert-X mice
% cell frequency
ns
ns
ns
NT-SA
SA-SA
CD4+ T cells
CD8+ T cells
B cells
b) IL-10 ELISPOT, IsdB stim.
IL-10 secreting cells/10^6
ns
***
****
PBS-B cells
SA-B cells
PBS-CD4+ T cells
SA-CD4+ T cells
c) IL-10 conc. post-vaccination, IsdB stim.
Conc. pg/ml
ns
****
***
SA-Alum
SA-AIsdB
B cells
CD4+ T cells
d) SA-CD4+ T cells → recipient with aIL10 +Vax
Transfer
WT recipient
SA-CD4+ T cells
aIL-10 Ab + vaccine
SA challenge
Spleen log10 CFU/ml
kidneys log10CFU/ml
Alum
AIsdB
IgG1
aIL-10
SA-CD4+ T cells
e) aCD25 or aCD69 Ab + vaccine
Spleen log10 CFU/ml
SA-Alum
SA-AIsdB
IgG1
aCD25
aCD69

Supplementary Fig. 3 CD4$^+$CD25$^+$ IL-10$^+$ T cells, induced by SA exposure, mediate blunting of IsdB vaccine efficacy

a) Percentage of splenic CD4$^+$ T, CD8$^+$ T and B220$^+$ B cells in naïve (n=5) or SA-exposed (n=7) Vert-X mice assessed as in **Supplementary Fig. 2c**.

b) Purified B or CD4$^+$ T cells (1x10^5) from PBS or SA-exposed (3x, 3x10^7CFU) WT mice were stimulated with IsdB antigen (10µg/ml) for 40 h, followed by detection of IL-10 by an ELISPOT assay (n=5 per group).

c) Pooled purified B (n=5) or CD4$^+$ T cells (n=5) from Alum or AIsdB SA-exposed vaccinated mice were stimulated with IsdB (10 µg/ml) for 60 h, followed by IL-10 measurement from supernatants.

d) CD4$^+$ T cells were isolated from SA-exposed mice and transferred into naïve mice. The recipient mice (n=17) were vaccinated in the presence of anti-IL10 Mab or isotype control (on day before and day of vaccination), then challenged with SA as in main **Fig. 1a.** For SA-CD4$^+$ T Alum, n= 16, and n=5 in AIsdB.

e) SA-exposed WT mice were depleted of either CD25$^+$ (n=9) or CD69$^+$ (n=8) T cells, vaccinated one day after, then SA challenged (n=8-9 per group). SA-Alum, n=8; SA-AIsdB (IgG1), n=9)

Data were from one to three independent experiments with each data point representing one mouse, except in **c**. The data is presented as mean ± SD of biological replicates., except in **c** (pooled data from five mouse represented as mean ± SD of three technical replicates). Data in **a-c** were analyzed by one-way ANOVA with Tukey`s posthoc test, while the data in **d-e** were analyzed by Kruskal-Wallis non-parametric one-way ANOVA test. *p<0.05, **p<0.01, ***p<0.001, ****p<0.0001. ns, non-significant. a, anti. SA, *Staphylococcus aureus.* Conc., concentration. WT, wild-type. Mouse image was created by BioRender (Created in BioRender. Hajam, I. (2024) https://BioRender.com/a18v205).

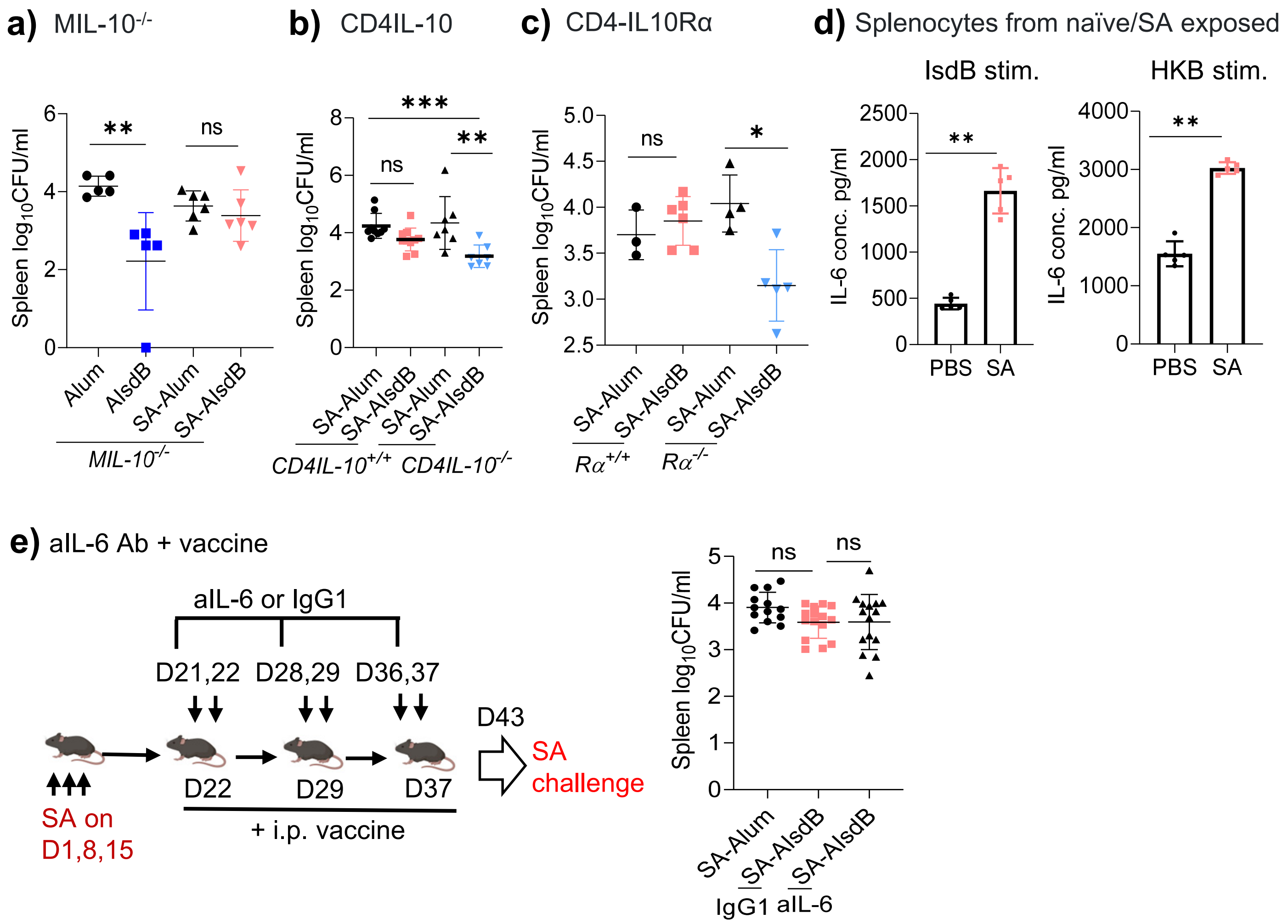
a) MIL-10-/-
Spleen log10CFU/ml
**
ns
Alum
AIsdB
SA-Alum
SA-AIsdB
MIL-10-/-
b) CD4IL-10
***
ns
**
CD4IL-10+/+
CD4IL-10-/-
c) CD4-IL10Rα
ns
*
Rα+/+
Rα-/-
d) Splenocytes from naïve/SA exposed
IsdB stim.
HKB stim.
IL-6 conc. pg/ml
PBS
SA
e) aIL-6 Ab + vaccine
aIL-6 or IgG1
D21,22
D28,29
D36,37
D43
SA challenge
SA on D1,8,15
D22
D29
D37
+ i.p. vaccine
IgG1
aIL-6

Supplementary Fig. 4 IL-10 released by $CD4^+$ T cells contribute directly to vaccine failure via IL-10Rα expressed on $CD4^+$ T cells.

a) Naïve or SA-exposed MIL-$10^{-/-}$ (IL$10^{flox/flox}$ x LysM^{cre}) were vaccinated i.p. with either Alum (naive, n=5; SA-exposed, n=6) or AIsdB (Naive, n=5; SA-exposed, n=6), then challenged with SA 7 dpv.

b) SA-exposed CD4IL-$10^{+/+}$ (or CD4IL-$10^{-/-}$ were vaccinated i.p. with either Alum (CD4IL-$10^{+/+}$ n=9; CD4IL$10^{-/-}$, n=7) or AIsdB (CD4IL-$10^{+/+}$,n=10; CD4IL$10^{-/-}$, n=7), then challenged with SA 7 dpv.

c) SA-exposed CD4IL10Rα$^{+/+}$ and CD4IL10Rα$^{-/-}$ were vaccinated i.p. with either Alum (CD4IL-10Rα$^{+/+}$n=3; CD4IL10Rα$^{-/-}$, n=4) or AIsdB (CD4IL-10Rα$^{+/+}$, n=6; CD4IL10Rα$^{-/-}$, n=5), then challenged with SA 7 dpv.

d) Splenocytes (pooled, n=5) from PBS- or SA-exposed **(**3 x 10^7CFU**)** WT mice were stimulated with either IsdB antigen (10 µg/ml) or HKB (1:10) *in vitro*. Cytokine IL-6 from supernatant was evaluated after 60h.

e) SA-exposed WT mice were treated with either isotype IgG1 control (n=15) or anti-IL-6 MAb (n=15) one day before and on the day of vaccination, then challenged with SA (n=13-15 per group). SA-Alum, n=13.

Data were from one to three independent experiments with each data point representing one mouse. The data is presented as mean ± SD of biological replicates., except in **d** (data is presented as mean ± SD of five technical replicates). The data in **a-c, e** were analyzed by Kruskal-Wallis non-parametric one-way ANOVA test, while the data in **d** was analyzed by two-tailed non-parametric Mann-Whitney T test. *p<0.05, **p<0.01, ***p<0.001, ns-non-significant. aIL-16, antiIL-6, dpv, days post-last vaccination. SA, *Staphylococcus aureus.* WT, wild-type. Source data are provided as Source Data File. Mouse image was created by BioRender (Created in BioRender. Hajam, I. (2024) https://BioRender.com/a18v205).

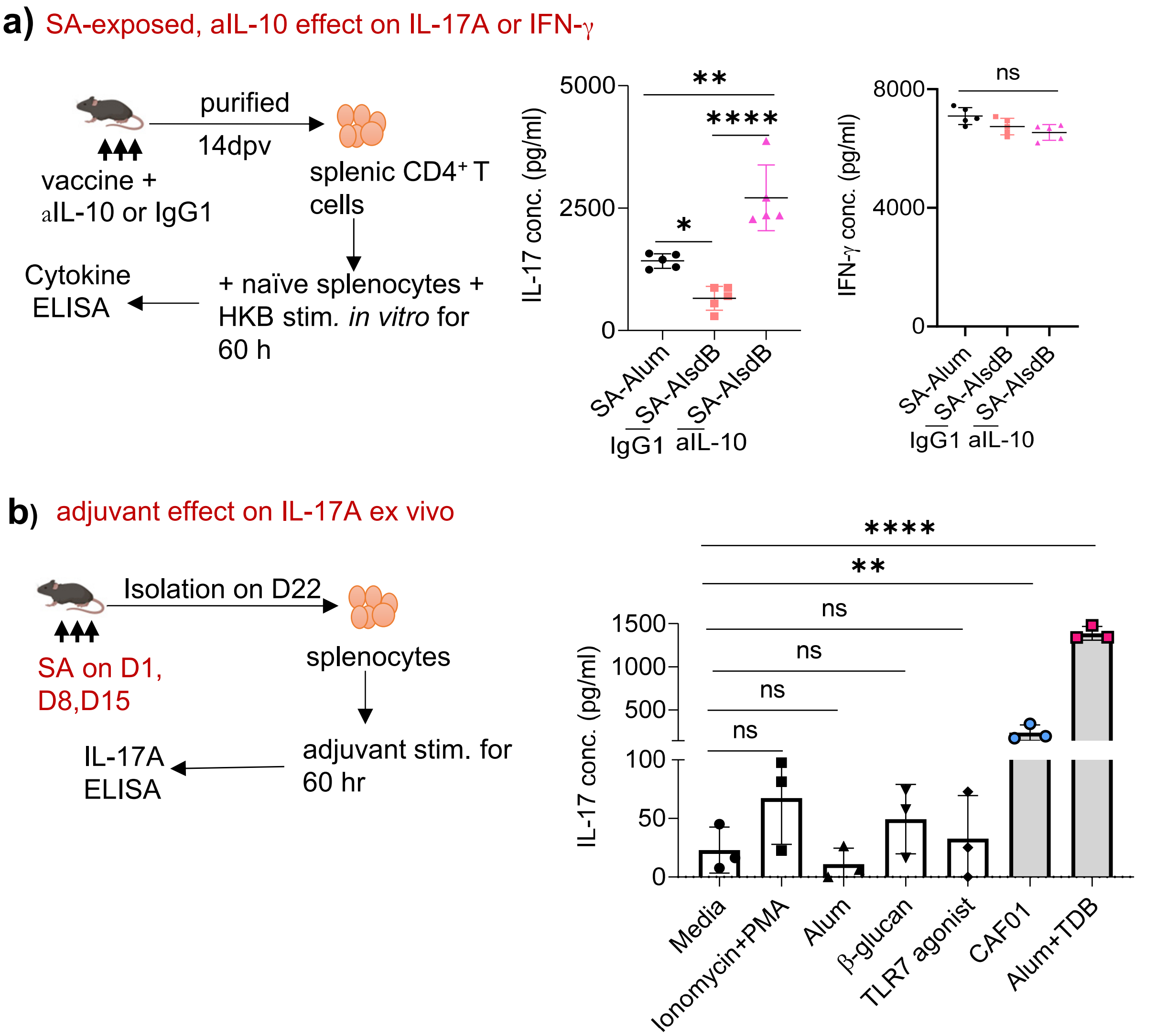

Supplementary Fig. 5 Effect of IL-10 expressing CD4⁺ T cell imprints on IL-17A, and the impact of adjuvants

a) SA-exposed WT mice were treated with either isotype IgG1 control or anti-IL-10 MAb one day before and on the day of AIsdB vaccination as in main Fig.2b. Splenic CD4⁺ T cells were purified 14 dpv and incubated with naïve splenocytes plus HKB (1:10). After 60h, supernatants were assayed for IL-17A and IFN-γ (n=5 per group).

b) Splenocytes were isolated from WT mice exposed to SA 7 days after the last SA exposure. The cells were stimulated with various adjuvants (10 µg/well) *in vitro* and IL-17A analysis was performed from supernatants after 60h (pooled, n=5 per group).

Data were from one experiment with each data point representing one mouse in **a** and each dot in **b** is a technical replicate. The data is presented as mean ± SD of biological replicates in **a** while in **b** data is presented a**s** mean ± SD of three technical replicates of one independent experiment**.** The data were analyzed by one-way ANOVA with Tukey`s post-hoc test. *p<0.05, **p<0.01, ****p<0.0001, ns-non-significant. HKB, heat-killed bacteria. aIL-10, antiIL-10. SA, *Staphylococcus aureus.* WT, wild-type. Source data are provided as Source Data File. Mouse image was created by BioRender (Created in BioRender. Hajam, I. (2024) https://BioRender.com/a18v205).

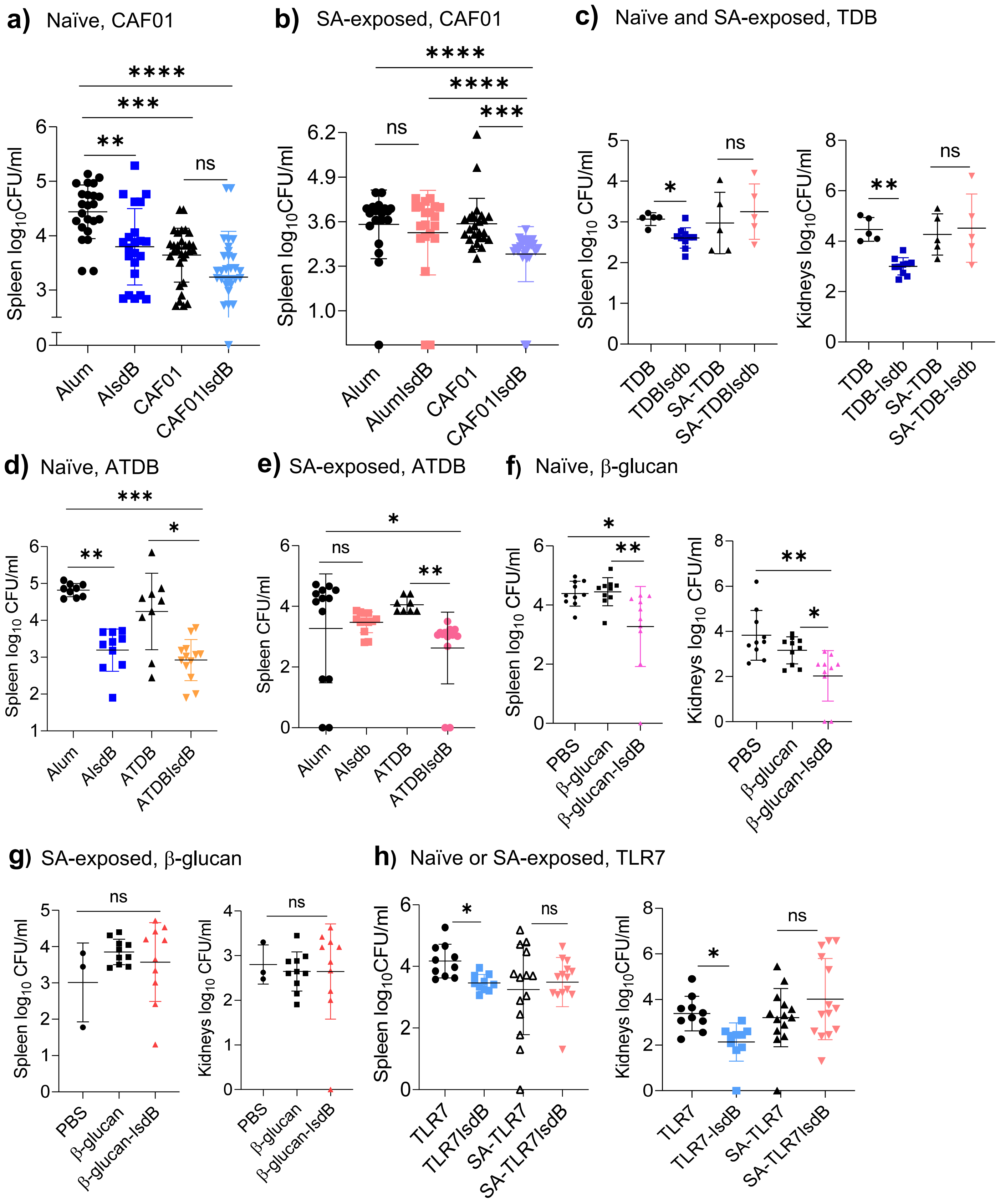
a) Naïve, CAF01
Spleen log10CFU/ml
Alum
AIsdB
CAF01
CAF01IsdB
****
***
**
ns
b) SA-exposed, CAF01
Spleen log10CFU/ml
Alum
AlumIsdB
CAF01
CAF01IsdB
****
****
***
ns
c) Naïve and SA-exposed, TDB
Spleen log10 CFU/ml
TDB
TDBIsdb
SA-TDB
SA-TDBIsdb
*
ns
Kidneys log10CFU/ml
TDB
TDB-Isdb
SA-TDB
SA-TDB-Isdb
**
ns
d) Naïve, ATDB
Spleen log10 CFU/ml
Alum
AIsdB
ATDB
ATDBIsdB
***
**
*
e) SA-exposed, ATDB
Spleen CFU/ml
Alum
AIsdb
ATDB
ATDBIsdB
*
ns
**
f) Naïve, β-glucan
Spleen log10 CFU/ml
PBS
β-glucan
β-glucan-IsdB
*
**
Kidneys log10 CFU/ml
PBS
β-glucan
β-glucan-IsdB
**
*
g) SA-exposed, β-glucan
Spleen log10 CFU/ml
PBS
β-glucan
β-glucan-IsdB
ns
Kidneys log10 CFU/ml
PBS
β-glucan
β-glucan-IsdB
ns
h) Naïve or SA-exposed, TLR7
Spleen log10CFU/ml
TLR7
TLR7IsdB
SA-TLR7
SA-TLR7IsdB
*
ns
Kidneys log10CFU/ml
TLR7
TLR7-IsdB
SA-TLR7
SA-TLR7IsdB
*
ns

Supplementary Fig. 6 High potency IL-17A adjuvants restore vaccine protection in SA-exposed mice.

a, b) Naïve (**b**) or SA-exposed WT mice (**c**) were vaccinated i.p. with Alum (n=23 in naïve and n=19 in SA-exposed), AIsdB (n=22 in naïve and n=20 in SA-exposed), CAF01 (n=30 in naïve and n=25 in SA-exposed) or CAF01IsdB (n=32 in naïve and n=26 in SA-exposed, followed by SA challenge 14 dpv.

c) Naïve and SA-exposed WT mice were vaccinated i.p. with TDB alone (n=5 in naïve and n=5 in SA-exposed) or TDBIsdB (n=10 in naïve and n=5 in Sa-exposed), then challenged with SA 14 dpv (n=5-10 per group).

d, e) Naïve (**d)** or SA-exposed WT mice (**e**) were vaccinated i.p. with Alum (n=9 in naïve and n=13 in SA-exposed), AIsdB (n=10 in naïve and n=13 in SA-exposed), AlumTDB (ATDB) (n=9 in naïve and n=8 in SA-exposed) or ATDBIsdB (n=13 in naïve and n=13 in SA-exposed), followed by SA challenge 14 dpv.

f, g) Naïve (**f,** n=10/group) or SA-exposed WT mice (**g)** were vaccinated i.p. with β-glucan (n=10 in SA-exposed) or β-glucanIsdB (n=10 in SA-exposed), then SA challenged on 14 dpv. PBS, n=3 in **g.**.

h) Naïve (n=10/group) or SA-exposed WT mice (n=14/group) were vaccinated i.p. with TLR7 agonist or or TLR7 agonist plus IsdB or , followed by SA challenge on 14 dpv.

Data were from one to 4 independent experiments with each data point representing one mouse. The data is presented as mean ± SD of biological replicates**.** The data were analyzed by Kruskal-Wallis non-parametric one-way ANOVA test. *p<0.05, **p<0.01, ***p<0.001, ****p<0.0001. ns-non-significant. SA, dpv, days post-last vaccination. *Staphylococcus aureus*. WT, wild-type. Source data are provided as Source Data File.

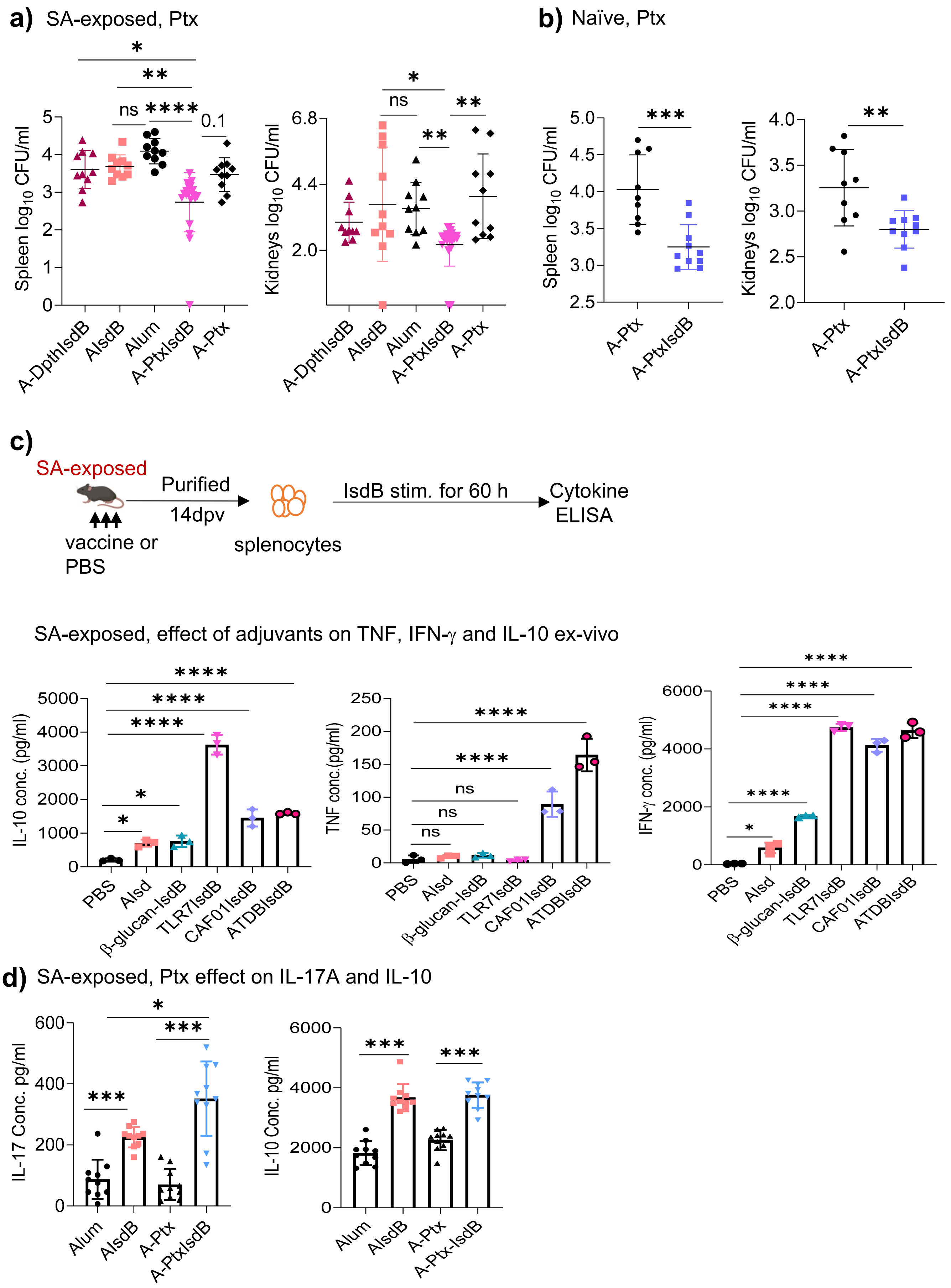
a)
SA-exposed, Ptx
Spleen log10 CFU/ml
Kidneys log10 CFU/ml
A-DpthIsdB
AIsdB
Alum
A-PtxIsdB
A-Ptx
ns
0.1
b)
Naïve, Ptx
Spleen log10 CFU/ml
Kidneys log10 CFU/ml
A-Ptx
A-PtxIsdB
c)
SA-exposed
vaccine or
PBS
Purified
14dpv
splenocytes
IsdB stim. for 60 h
Cytokine
ELISA
SA-exposed, effect of adjuvants on TNF, IFN-γ and IL-10 ex-vivo
IL-10 conc. (pg/ml)
TNF conc.(pg/ml)
IFN-γ conc. (pg/ml)
PBS
AIsd
β-glucan-IsdB
TLR7IsdB
CAF01IsdB
ATDBIsdB
ns
d)
SA-exposed, Ptx effect on IL-17A and IL-10
IL-17 Conc. pg/ml
IL-10 Conc. pg/ml
Alum
AIsdB
A-Ptx
A-PtxIsdB
A-Ptx-IsdB

Supplementary Fig. 7 IL-17A responses correlate with vaccine protection in SA-exposed mice

a) SA-exposed WT mice were vaccinated i.p. with Alum (n=10), AIsdB (n=10), Alum-diphtheria toxoid-IsdB (A-DpthIsdB, n=10)) or A-PtxIsdB (n-20), then challenged with SA 14 dpv.

b) Naïve WT mice were vaccinated i.p. with A-Ptx (n=9) or A-PtxIsdB (n=10), then SA challenged 14 dpv.

c) SA-exposed (3x, $3x10^7$ CFU) WT mice were vaccinated i.p. with either PBS or IsdB plus adjuvant. Splenocytes (pooled from three mouse) purified on 14 dpv were stimulated with IsdB (10 µg/ml), and culture supernatants were analyzed for IL-17A after 60h.

d) SA-exposed WT mice were vaccinated i.p. with Alum, AIsdB, Alum-pertussis toxoid (A-Ptx) or A-PtxIsdB. Splenocytes purified on 14 dpv were stimulated with IsdB (10 µg/ml), and culture supernatants were analyzed for IL-17A after 60h (n=10 per group).

Data were from one to 3 independent experiments with each data point representing one mouse, except in **c**. The data is presented as mean ± SD of biological replicates, except in **c** (data is presented as mean ± SD of three technical replicates of one independent experiment and repeated twice with similar results). The data in **a,d** was analyzed by Kruskal-Wallis non-parametric one-way ANOVA test, data in **b** by two-tailed non-paired Mann-Whitney T test, while the data in **c** by one-way ANOVA with Tukey`s post-hoc test, *p<0.05, **p<0.01, ***p<0.001, ****p<0.0001 ns-non-significant. dpv, days post-last vaccination. Ptx, pertussis toxoid. SA, *Staphylococcus aureus*. WT, wild-type. Source data are provided as Source Data File. Mouse image was created by BioRender (Created in BioRender. Hajam, I. (2024) https://BioRender.com/a18v205).

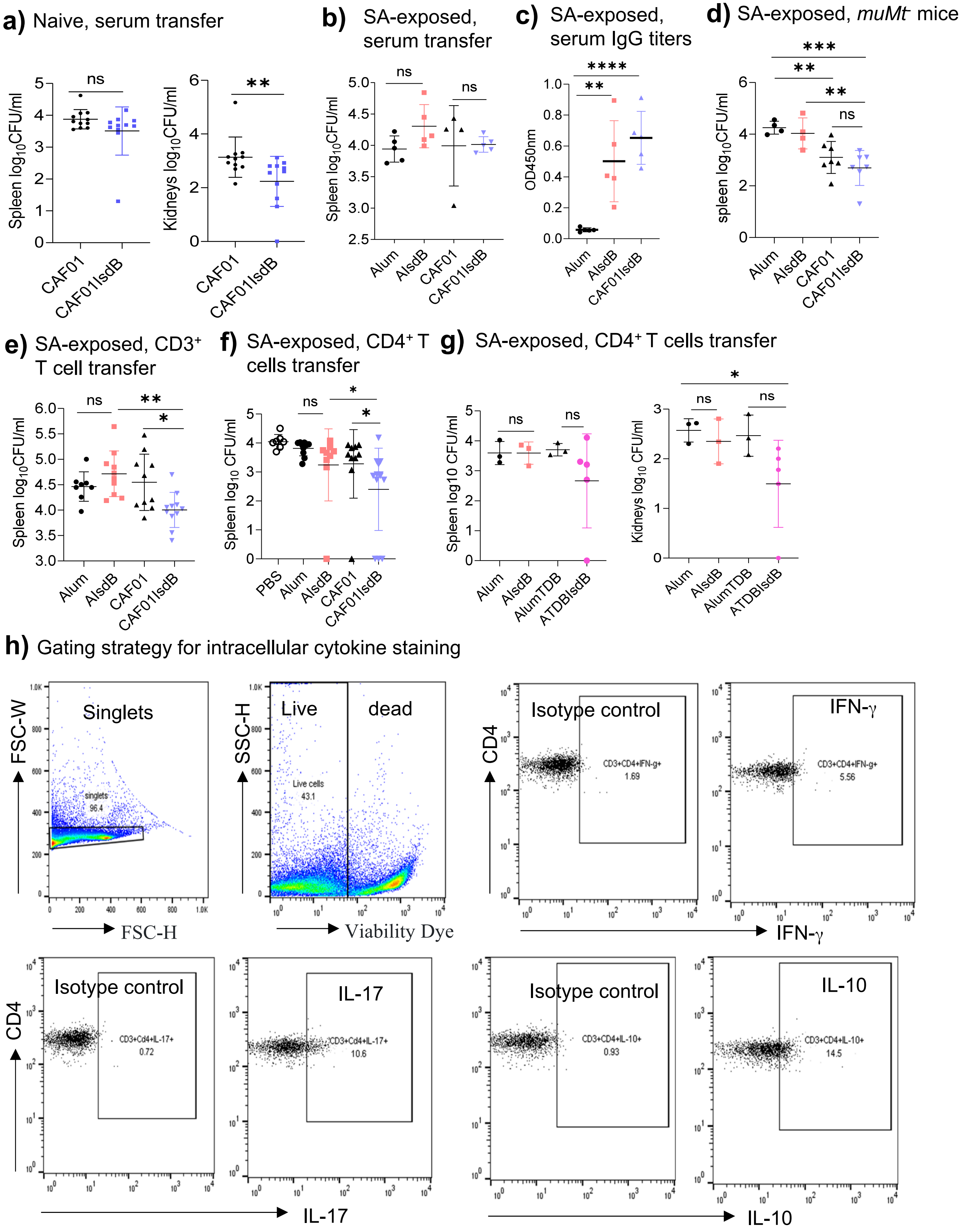
a) Naive, serum transfer
Spleen log10CFU/ml
Kidneys log10CFU/ml
CAF01
CAF01IsdB
ns
**
b) SA-exposed, serum transfer
Spleen log10CFU/ml
Alum
AIsdB
CAF01
CAF01IsdB
c) SA-exposed, serum IgG titers
OD450nm
****
d) SA-exposed, muMt mice
spleen log10CFU/ml
***
e) SA-exposed, CD3+ T cell transfer
f) SA-exposed, CD4+ T cells transfer
Spleen log10 CFU/ml
PBS
g) SA-exposed, CD4+ T cells transfer
Spleen log10 CFU/ml
Kidneys log10 CFU/ml
AlumTDB
ATDBIsdB
h) Gating strategy for intracellular cytokine staining
Singlets
FSC-W
FSC-H
singlets
96.4
Live
dead
SSC-H
Viability Dye
Live cells
43.1
Isotype control
IFN-γ
CD4
CD3+CD4+IFN-g+
1.69
5.56
IL-17
CD3+Cd4+IL-17+
0.72
10.6
IL-10
CD3+CD4+IL-10+
0.93
14.5

Supplementary Fig. 8 Th17 adjuvants induce vaccine protection in SA-exposed mice that is CD4$^+$ T cell dependent.

a, b) Serum (150 µl) collected 14dpv of naïve (**a**, n=10 per group) or SA-exposed (**b**, n=5 per group, except in Alum where n=4) WT mice was transferred i.v. into naive recipient mice. After 20h, the mice were challenged with SA.

c) IgG titers (diluted 1:100000) of serum collected 14 dpv from SA-exposed vaccinated mice (n=5 per group).

d) SA-exposed *muMT*$^{-}$ mice were vaccinated i.p. with Alum (n=3), AIsdB (n=4), CAF01 (n=7) or CAF01IsdB (n=7), then SA challenged 14 dpv.

e, f) SA-exposed WT mice were vaccinated i.p. with Alum (n=8 in **e** and n=10 in **f**), AIsdB (n=10 in **e** and n=9 in **f**), CAF01 (n=8 in **e,f**) or CAF01IsdB (n=11 in **e** and n=13 in **f**). 14 dpv, splenic CD3$^+$ T cells (**e**) or CD4$^+$ T cells (**f**) were isolated and i.v. transferred into naïve WT mice, followed by SA challenge of the recipient mice after 20 h. PBS, n=7 in **f**.

g) SA-exposed WT mice were vaccinated i.p. with Alum (n=3), AIsdB (n=3), ATDB (n=3 or ATDBIsdB (n=4). 14 dpv, splenic CD4$^+$ T cells were isolated and transferred i.v. into naïve WT mice, followed by SA challenge of the mice after 20 h.

h) Gating strategy for the detection of intracellular cytokines.

Data were from one to two independent experiments with each data point representing one mouse. The data is presented as mean ± SD of biological replicates**.** The data were analyzed by one-way ANOVA with Tukey`s post-hoc test. *p<0.05, **p<0.01, ***p<0.001, ****p<0.0001, ns-non-significant. SA, dpv, days post-last vaccination. *Staphylococcus aureus*. WT-wild-type. Source data are provided as Source Data File.

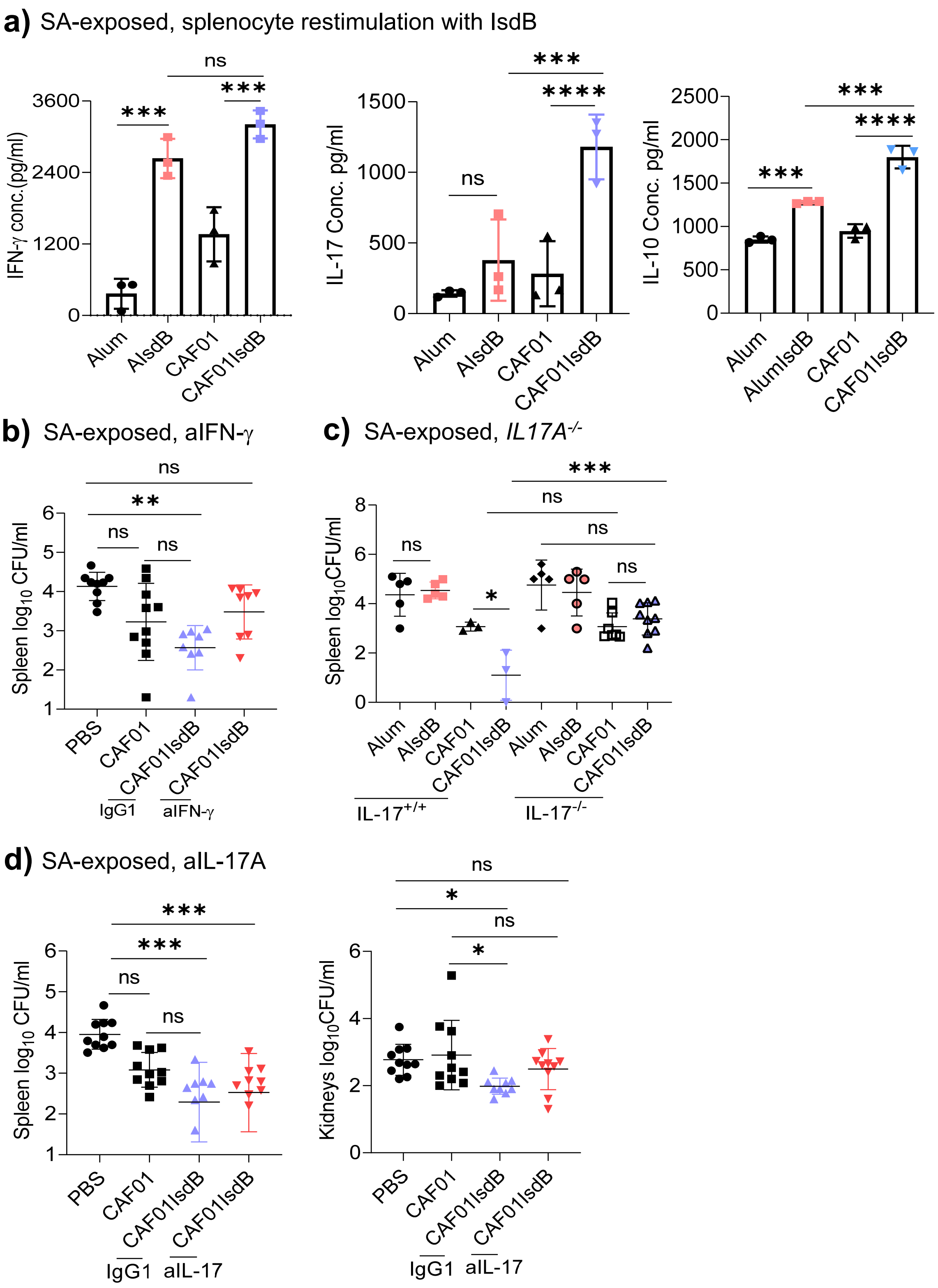
a) SA-exposed, splenocyte restimulation with IsdB
IFN-γ conc.(pg/ml)
IL-17 Conc. pg/ml
IL-10 Conc. pg/ml
Alum
AIsdB
AlumIsdB
CAF01
CAF01IsdB
ns
***
****
b) SA-exposed, aIFN-γ
Spleen log10 CFU/ml
PBS
IgG1
aIFN-γ
**
c) SA-exposed, IL17A-/-
Spleen log10CFU/ml
IL-17+/+
IL-17-/-
*
d) SA-exposed, aIL-17A
Kidneys log10CFU/ml
aIL-17

Supplementary Fig. 9 CAF01 adjuvant induces IL-17A and IFN-γ dependent protection in SA-exposed mice.

a) SA-exposed WT mice were vaccinated i.p. with Alum, AIsdB, CAF01 or CAF01IsdB. 14 dpv, splenocytes ($1x10^6$, n=5, pooled) were isolated and stimulated with IsdB antigen (10 µg/ml) for 60 h, and cytokines from culture supernatants were analyzed. Data is presented as mean ± SD of three technical replicates of one experiment and the experiment was repeated twice.

b) SA-exposed WT mice were vaccinated i.p. with CAF01 (n=10) or CAF01IsdB. 30 dpv, the mice were treated with an isotype IgG1 control (n=8) or anti-IFN-γ MAb (n=8) one day before and on the day of SA challenge (n=8-10 per group). PBS, n=9.

c) SA-exposed WT or $IL-17^{-/-}$ mice were vaccinated i.p. with Alum (n=5), AIsdB (n=5), CAF01 (n=3 in WT and n=7 in ko) or CAF01IsdB (n=3 in WT and n=9 in ko), then challenged with SA 14 dpv.

d) SA-exposed WT mice were vaccinated i.p. withCAF01 or CAF01IsdB. 30 dpv, the mice were treated with an isotype IgG1 control or anti-IL-17A MAb one day before and on the day of SA challenge (n=10 per group, except in IgG1 control where n=9).

Data were from one to two independent experiments with each data point representing one mouse in **b-d.** The data is presented as mean ± SD of biological replicates**,** except in **a.** The data in **a, c** was analyzed by one-way ANOVA test with Tukey`s post-hoc test, while the data in **b, d** by Kruskal-Wallis non-parametric one-way ANOVA test. *p<0.05, **p<0.01, ***p<0.001, ****p<0.0001. ns-non-significant. a, anti. SA*,* dpv*,* days post-last vaccination. *Staphylococcus aureus.* WT*,* wild-type*.* Source data are provided as Source Data File.

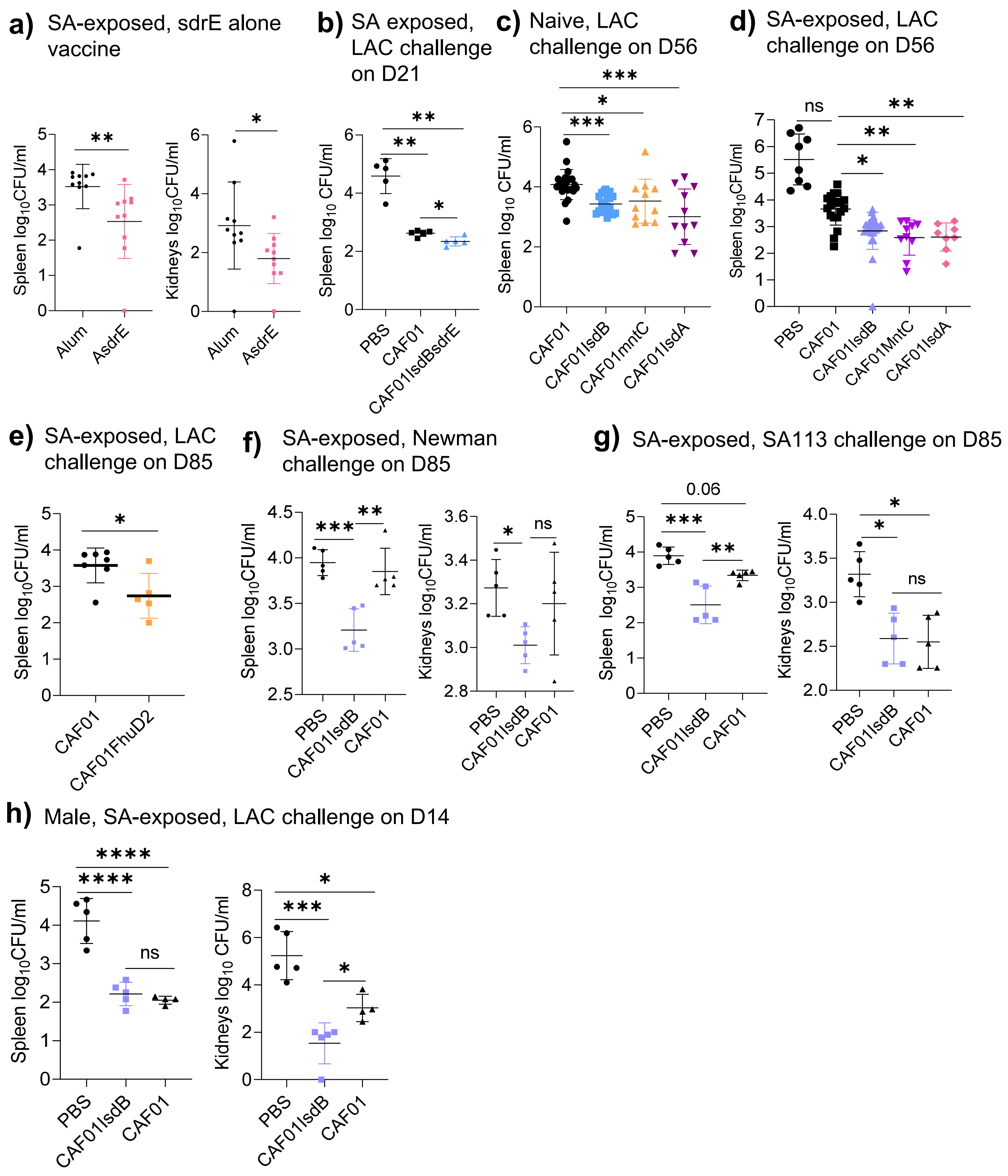

a) SA-exposed, sdrE alone vaccine
Spleen log10CFU/ml
Kidneys log10CFU/ml
Alum
AsdrE
b) SA exposed, LAC challenge on D21
Spleen log10 CFU/ml
PBS
CAF01
CAF01IsdBsdrE
c) Naive, LAC challenge on D56
Spleen log10 CFU/ml
CAF01
CAF01IsdB
CAF01mntC
CAF01IsdA
d) SA-exposed, LAC challenge on D56
Spleen log10CFU/ml
ns
PBS
CAF01
CAF01IsdB
CAF01MntC
CAF01IsdA
e) SA-exposed, LAC challenge on D85
Spleen log10CFU/ml
CAF01
CAF01FhuD2
f) SA-exposed, Newman challenge on D85
Spleen log10CFU/ml
Kidneys log10CFU/ml
ns
PBS
CAF01IsdB
CAF01
g) SA-exposed, SA113 challenge on D85
Spleen log10CFU/ml
Kidneys log10CFU/ml
0.06
ns
PBS
CAF01IsdB
CAF01
h) Male, SA-exposed, LAC challenge on D14
Spleen log10CFU/ml
Kidneys log10 CFU/ml
ns
PBS
CAF01IsdB
CAF01

Supplementary Fig. 10 CAF01 adjuvant induces long lasting protection against multiple strains of SA.

a) SA-exposed WT mice were vaccinated i.p. with Alum or combination AIsdBsdrE vaccine, then SA challenged (n=10 per group).

b) SA-exposed WT mice were vaccinated i.p. with PBS, CAF01 or CAF01IsdBsdrE, then challenged with SA 21 dpv (n=5 per group).

c, d) Naïve (**c**) or SA-exposed WT mice (**d,** n=8-25) were vaccinated i.p. with PBS (n=8), CAF01 (n=22 in **c** and 19 in **d**), CAF01IsdB (n=23 in **c** and 25 in **d**), CAF01mntC (n=11 in **c** and 10 in **d**) or CAF01IsdA (n=11 in **c** and 8 in **d**), followed by SA challenge 56 dpv.

e) SA-exposed WT mice were vaccinated i.p. with CAF01 (n=7) or CAF01FhuD2 (n=5), followed by SA challenge 85 dpv.

f, g) SA (Newman (**f**) or SA113 (**g**)-exposed WT mice (3x, $3x10^7$ CFU) were vaccinated i.p. with CAF01 (n=5) or CAF01IsdB (n=5), followed by challenge with the same SA strain on 85 dpv.

h) SA-exposed WT male mice were vaccinated i.p. with CAF01 (n=4) or CAF01IsdB (n=5), then SA challenge 14 dpv. PBS, n=5.

Data were from one to four independent experiments with each data point representing one mouse. The data is presented as mean ± SD of biological replicates**.** The data in **a,e** was analyzed by two tailed non-parametric unpaired Mann-Whitney T test, data in **b,f-h** by one-way ANOVA test, while the data in **c-d** by Kruskal-Wallis non-parametric one-way ANOVA test. *p<0.05, **p<0.001, ***p<0.0001, ns-non-significant. D, day. SA, *Staphylococcus aureus.* Source data are provided as Source Data File.

Supplementary Table 1 Antibody concentration used in flow cytometry.

Antibody	Company	Catalog number	Dilution used for staining cells
PE anti-mouse/human CD45R/B220	Biolegend, USA	103208	1:100
APC anti-mouse CD3	Biolegend, USA	100236	1:100
Pacific Blue anti-mouse CD4	Biolegend, USA	100427	1:100
PerCP/Cyanine5.5 anti-mouse CD4	Biolegend, USA	100434	1:100
PerCP/Cyanine5.5 anti-mouse CD8a	Biolegend, USA	100733	1:100
PE anti-mouse CD69	Biolegend, USA	104508	1:100
Pacific Blue anti-mouse CD25	Biolegend, USA	102022	1:100
PE/Cyanine7 anti-mouse IL-10	Biolegend, USA	505026	1:50
PE anti-mouse IL-17A	Biolegend, USA	506903	1:50
PerCP/Cyanine5.5 anti-mouse IFN-γ	Biolegend, USA	505822	1:50
PE/Cyanine7 Rat IgG2b	Biolegend, USA	400617	1:50
PE Rat IgG1	Biolegend, USA	400407	1:50
PerCP/Cyanine5.5 Rat IgG1	Biolegend, USA	400425	1:50
