# Supplementary material for: Pathobiont-induced suppressive immune imprints thwart T cell vaccine responses
Source: Nat Commun. 2024 Dec 16;15:10335. doi: 10.1038/s41467-024-54644-w (PMC11649901; doi:10.1038/s41467-024-54644-w)
Supplement: Supplementary file 2 — Reporting Summary [file 41467_2024_54644_MOESM2_ESM.pdf]

nature portfolio

Corresponding author(s): George Y. Liu

Last updated by author(s): 2024/10/24

Reporting Summary

Nature Portfolio wishes to improve the reproducibility of the work that we publish. This form provides structure for consistency and transparency in reporting. For further information on Nature Portfolio policies, see our Editorial Policies and the Editorial Policy Checklist.

Statistics

For all statistical analyses, confirm that the following items are present in the figure legend, table legend, main text, or Methods section.

n/a	Confirmed	
☐	☒	The exact sample size (*n*) for each experimental group/condition, given as a discrete number and unit of measurement
☐	☒	A statement on whether measurements were taken from distinct samples or whether the same sample was measured repeatedly
☐	☒	The statistical test(s) used AND whether they are one- or two-sided<br>*Only common tests should be described solely by name; describe more complex techniques in the Methods section.*
☒	☐	A description of all covariates tested
☐	☒	A description of any assumptions or corrections, such as tests of normality and adjustment for multiple comparisons
☐	☒	A full description of the statistical parameters including central tendency (e.g. means) or other basic estimates (e.g. regression coefficient) AND variation (e.g. standard deviation) or associated estimates of uncertainty (e.g. confidence intervals)
☐	☒	For null hypothesis testing, the test statistic (e.g. *F*, *t*, *r*) with confidence intervals, effect sizes, degrees of freedom and *P* value noted<br>*Give P values as exact values whenever suitable.*
☒	☐	For Bayesian analysis, information on the choice of priors and Markov chain Monte Carlo settings
☒	☐	For hierarchical and complex designs, identification of the appropriate level for tests and full reporting of outcomes
☒	☐	Estimates of effect sizes (e.g. Cohen's *d*, Pearson's *r*), indicating how they were calculated

Our web collection on statistics for biologists contains articles on many of the points above.

Software and code

Policy information about availability of computer code

Data collection	Flow cytometric data was collected using BD FACSCanto™ II Flow Cytometry
Data analysis	GraphPad Prism 8.0 (GraphPad Inc., San Diego, CA)<br>FlowJo v.10 software.(Ashland, OR 97520, USA)

For manuscripts utilizing custom algorithms or software that are central to the research but not yet described in published literature, software must be made available to editors and reviewers. We strongly encourage code deposition in a community repository (e.g. GitHub). See the Nature Portfolio guidelines for submitting code & software for further information.

Data

Policy information about availability of data

All manuscripts must include a data availability statement. This statement should provide the following information, where applicable:

- Accession codes, unique identifiers, or web links for publicly available datasets
- A description of any restrictions on data availability
- For clinical datasets or third party data, please ensure that the statement adheres to our policy

All data are included in the Supplementary Information or available from the authors, as are unique reagents used in this Article. The raw numbers for charts and graphs are available in the Source Data file whenever possible.

April 2023

Research involving human participants, their data, or biological material

Policy information about studies with human participants or human data. See also policy information about sex, gender (identity/presentation), and sexual orientation and race, ethnicity and racism.

Reporting on sex and gender	Not relevant
Reporting on race, ethnicity, or other socially relevant groupings	Not relevant to this study
Population characteristics	Not relevant to this study
Recruitment	Not relevant to this study
Ethics oversight	Not relevant to this study

Note that full information on the approval of the study protocol must also be provided in the manuscript.

Field-specific reporting

Please select the one below that is the best fit for your research. If you are not sure, read the appropriate sections before making your selection.

☒ Life sciences ☐ Behavioural & social sciences ☐ Ecological, evolutionary & environmental sciences

For a reference copy of the document with all sections, see nature.com/documents/nr-reporting-summary-flat.pdf

Life sciences study design

All studies must disclose on these points even when the disclosure is negative.

Sample size	No Statistical predetermination of sample size was performed. Sample sizes were selected based on pilot experiments or based on other published work that gave reliable statistical results.
Data exclusions	Outlier analysis was performed by GraphPad Prism version 8. One outlier mouse was removed from Fig.1b, 1h, 2j, 4d, and 2 mice from Fig.1f
Replication	The number of experimental replicates are indicated in the respective figure legends. Some of the experiments were performed by two independent researchers as previously mentioned in the paper.
Randomization	Age-matched animals were randomly assigned to experimental groups, and assigned to either PBS, adjuvant or adjuvant plus vaccine group.
Blinding	Experiments were not blinded as animals were either giving PBS, adjuvant or adjuvant plus vaccine antigen, then protection measured based on the bacterial burden in organs.

Reporting for specific materials, systems and methods

We require information from authors about some types of materials, experimental systems and methods used in many studies. Here, indicate whether each material, system or method listed is relevant to your study. If you are not sure if a list item applies to your research, read the appropriate section before selecting a response.

Materials & experimental systems

n/a	Involved in the study	
☐	☒	Antibodies
☒	☐	Eukaryotic cell lines
☒	☐	Palaeontology and archaeology
☐	☒	Animals and other organisms
☒	☐	Clinical data
☒	☐	Dual use research of concern
☒	☐	Plants

Methods

n/a	Involved in the study	
☒	☐	ChIP-seq
☐	☒	Flow cytometry
☒	☐	MRI-based neuroimaging

Antibodies

Antibodies used	PE anti-mouse/human CD45R/B220 (Catalog No.#103208;1:100 dilution used), APC anti-mouse CD3 (Catalog No.#100236;1:100 dilution used), Pacific Blue™ anti-mouse CD4 (Catalog No.#100427;1:100 dilution used), PerCP/Cyanine5.5 anti-mouse CD4 (Catalog No.#100434;1:100 dilution used), PerCP/Cyanine5.5 anti-mouse CD8a (Catalog No.#100733;1:100 dilution used), PE anti-mouse CD69 (Catalog No.#104508;1:100 dilution used), Pacific Blue™ anti-mouse CD25 (Catalog No.#102022;1:100 dilution used), PE/

Cyanine7 anti-mouse IL-10 (Catalog No.#505026;1:50dilution used), PE anti-mouse IL-17A (Catalog No.#506903;1:50dilution used), PerCP/Cyanine5.5 anti-mouse IFN-γ (Catalog No.#505822;1:50 dilution used), PE/Cyanine7 Rat IgG2b (Catalog No.#400617;1:50 dilution used), PE Rat IgG1 (Catalog No.#400407;1:50dilution used), and PerCP/Cyanine5.5 Rat IgG1 (Catalog No.#400425;1:50 dilution used).

InVivoMAb anti-mouse IL-6 (Catalog #BE0046, BioXCell, USA; 25 µg/mouse), InVivoMAb anti-mouse IL-10 (Catalog #BE0049, BioXCell, USA; 25 µg/mouse), InVivoMAb rat IgG1 isotype control (Catalog #BE0088, BioXCell, USA; 25 µg/mouse).
InVivoMAb anti-mouse IFN-γ (Catalog #BE0054, BioXCell, USA; 100 µg/mouse), isotype control (rat IgG1, Catalog #BE0088; 100 µg/mouse). InVivoMAb anti-mouse IL-17A (Catalog # BE0173, BioXCell, USA; 100 µg/mouse), isotype control (mouse IgG1, Catalog # BE0083; 100 µg/mouse).

Validation

All the antibodies were validated by the vendors (Biolegend and Bio X cell).

Animals and other research organisms

Policy information about studies involving animals; ARRIVE guidelines recommended for reporting animal research, and Sex and Gender in Research

Laboratory animals

All mice (Mus musculus) used in this study were 6-8 weeks old, kept in filler-top cages with access to food pellet and water under controlled ambient temperature (20-22 degrees Celsius) and relative humidity (30-70%), 12 h light/12 h dark cycle.

Wild animals

Not applicable

Reporting on sex

Both females and males were used.

Field-collected samples

Not applicable

Ethics oversight

As described in the `Ethics Declarations` section, animal experiments/housing were conducted under the UC San Diego approved IRB protocol S18200, and in accordance with the rules and regulations of the Institutional Animal Care and Use Committee.

Note that full information on the approval of the study protocol must also be provided in the manuscript.

Plants

Seed stocks

Not applicable

Novel plant genotypes

Not applicable

Authentication

Not applicable

Flow Cytometry

Plots

Confirm that:

- [x] The axis labels state the marker and fluorochrome used (e.g. CD4-FITC).
- [x] The axis scales are clearly visible. Include numbers along axes only for bottom left plot of group (a 'group' is an analysis of identical markers).
- [x] All plots are contour plots with outliers or pseudocolor plots.
- [x] A numerical value for number of cells or percentage (with statistics) is provided.

Methodology

Sample preparation

Spleens were aseptically collected from vaccinated groups and homogenized in sterile PBS (pH 7.4). Cells were centrifuged at 400g for 5 min, followed by RBC lysis before resuspension of splenocytes in PBS. The cells (2x106) were stained with Fixable Viability Dye eFluor™ 780 (Catalog No. 65-0865-14, eBiosceince) on ice for 30 min, followed by washing with the FACS buffer (2%FBS in PBS). Then cells were incubated with FC block TruStain FcX™, Catalog No.# 101320, Biolegend) for 10 min, followed by surface staining with fluorescently labelled Abs against CD3, CD4, CD8, CD25, CD69 and B220 on ice for 30 min. The cells were washed and resuspended in FACS buffer. For intracellular cytokine staining, surface antibody staining is followed by fixation (IC Fixation Buffer, Catalog No. # 50-112-9058, eBiosceince™) at room temperature for 20 min, then washed with a permeabilization buffer (Catalog. No.# 421002, Biolegend, USA) and incubation with fluorescently labelled antibodies against IFN-γ, IL-17A and IL-10 in permeabilization buffer for 1 h. Cells were washed and resuspended in FACS buffer, and run on an

	BD FACSCanto™ II Flow Cytometry. The data was analyzed with FlowJo v.10 software.
Instrument	BD FACSCanto™ II Flow Cytometry
Software	FlowJo v.10 software (Ashland, OR 97520, USA)
Cell population abundance	No cell sorting was done.
Gating strategy	First singlet analysis was performed based on the FSC-W and FSC-H characteristics. Then dead cells were excluded from live cells using SSC-H vs viability dye. Then lymphocytes were gated and from lymphocytes, cells were selected based on the expressions of CD markers. For detection of intracellular cytokines, first CD3CD4 population was selected then IFN-γ, IL-17 or IL-0 based on the expression. Isotype controls were included in the intracellular cytokine staining.

☒ Tick this box to confirm that a figure exemplifying the gating strategy is provided in the Supplementary Information.
